# Discrimination of Frailty Phenotype by Kinect^TM^-Based Stepping Parameters

**DOI:** 10.14283/jarlife.2023.17

**Published:** 2023-12-20

**Authors:** Y. Osuka, N. Takeshima, N. Kojima, T. Kohama, E. Fujita, M. Kusunoki, Y. Kato, W.F. Brechue, H. Sasai

**Affiliations:** 1 Department of Frailty Research, Center for Gerontology and Social Science, Research Institute, National Center for Geriatrics and Gerontology, Aichi, Japan; 2 Research Team for Promoting Independence and Mental Health, Tokyo Metropolitan Institute for Geriatrics and Gerontology, Tokyo, Japan; 3 Department of Health and Sports Sciences, Asahi University, Gifu, Japan; 4 Faculty of Biology-Oriented Science and Technology, Kindai University, Wakayama, Japan; 5 Department of Sports and Life Science, National Institute of Fitness and Sports in Kanoya, Kagoshima, Japan; 6 Department of Physical Therapy, Nagoya Women’s University, Aichi, Japan; 7 Department of Physiology, Kirksville College of Osteopathic Medicine, A.T. Still University of Health Sciences, Missouri, USA

**Keywords:** aging, digital biomarker, frailty, mobility, Kinect^TM^

## Abstract

**Background:**

Frailty increases the risk of falling, hospitalization, and premature death, necessitating practical early-detection tools.

**Objectives:**

To examine the discriminative ability of Kinect^TM^-based stepping parameters for identifying frailty phenotype

**Design:**

Population-based cross-sectional study

**Setting:**

Eighteen neighborhoods near Tokyo Metropolitan Institute for Geriatrics and Gerontology, Itabashi, Tokyo, Japan.

**Participants:**

In total, 563 community-dwelling older adults aged ≥75 years without mobility limitations, neurological disease, or dementia were included.

**Measurements:**

Step number (SN) and knee total movement distance (KMD) during a 20-s stepping test were evaluated using the Kinect^TM^ infrared depth sensor.

**Results:**

The number (%) of participants with frailty were 51 (9.1). The area under the receiver operating characteristic curves (95% confidence interval) of a parameter consisting of SN and KMD for frailty was 0.72 (0.64, 0.79).

**Conclusions:**

Stepping parameters evaluated using Kinect^TM^ provided acceptable ability in identifying frailty phenotype, making it a practical screening tool in primary care and home settings.

## Introduction

Frailty increases the risk of falling, hospitalization, and premature death (1), necessitating practical early-detection tools (2). Fried’s frailty phenotype is the most frequently used assessment in clinical practice; however, its practicality is limited in busy or remote clinical settings because of space and time requirements and the necessity for trained health professionals (3). Inexpensive and easily available screening tools for frailty, utilizing digital health technology (e.g., smartphones and wearable devices) may help bridge this gap (4).

Digital parameters generated with the Kinect^TM^ infrared depth sensor provide a simple method for assessing postural control during standing (5) and specific spatiotemporal gait parameters (6). Recently, four physical performance tests (gait analysis, 30-s arm curl, 30-s chair sit-to-stand, and 2-min step) were quantified using skeletal data acquired from Kinect^TM^ sensors and a machine learning approach to identify the frailty level with 97.5% accuracy (7). These approaches provide accurate assessments but require significant human, place, and time resources to conduct four performance tests.

Mobility, defined as “the ability to move or walk freely and easily (8),” can be assessed through various performance tests, including 5-chair sit-to-stand, timed up-and-go [TUG], gait speed, and alternate stepping tests, indicating that these parameters help identify frailty risk (9). Among these, step-in-place tests, such as the alternative step test, are the most feasible for home or clinical settings, given their minimal space requirements. Our group has shown that Kinect^TM^ sensor-generated variables, such as number of steps and head movement during a 20-s step test (ST), identify older adults requiring care as defined by Japan’s long-term care insurance system (area under the receiver operating characteristic [AUC]: 0.76–0.82) (10). Thus, these stepping parameters evaluated using Kinect^TM^ technology may provide a more practical frailty screening tool in busy clinical or remote environments. However, the extent to which stepping performance assessed by Kinect^TM^ can identify frailty remains unclear. Thus, this study aimed to examine the discriminative ability of a 20-s ST using Kinect^TM^ to identify the frailty phenotype.

## Methods

### Setting and participants

This study involved community-dwelling older adults residing in 18 neighborhoods near the Tokyo Metropolitan Institute for Geriatrics and Gerontology, Itabashi, Tokyo, Japan. Itabashi Ward is a special ward located in the northwestern part of Tokyo. The population of Itabashi Ward in July 2019 was 570,522 (males: 279,919; 49.0%), while that of older adults aged ≥65 years was 131,167 (23.0%) (11).

**Figure 1.**
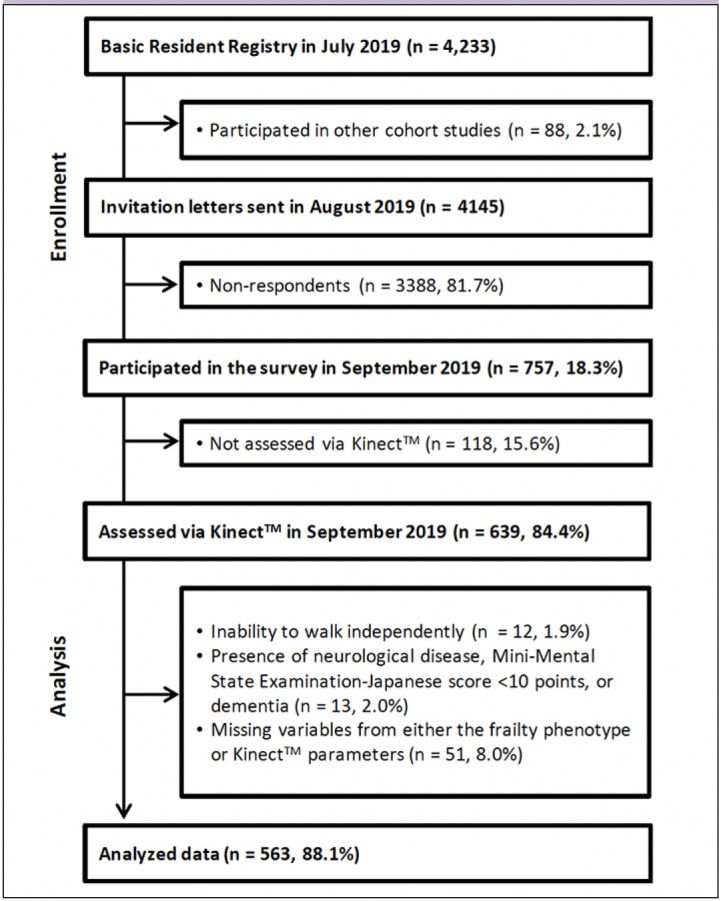
Study flow

Information regarding the names and addresses of all individuals aged 75–85 years, registered in the Basic Resident Registry for 18 areas, was collected, totaling 4,233 individuals. After excluding 88 participants who had joined other studies, invitations were extended to 4,145 potential candidates. Ultimately, 757 individuals participated in the study, with 639 undergoing a 20-s ST evaluation using Kinect^TM^. Exclusion criteria were as follows: 1) inability to walk independently (n=12, 1.9%); 2) presence of neurological disease, Mini-Mental State Examination-Japanese score <10 points, or dementia (n=13, 2.0%); and 3) missing variables from either the frailty phenotype or Kinect^TM^ parameters (n=51, 8.0%). Ultimately, 563 participants (88.1% of the original population) were included (Fig 1).

### Frailty phenotype

Frailty phenotype was assessed using the revised Japanese version of the Cardiovascular Health Study criteria (12). Frailty and prefrailty were defined as the presence of ≥3 and 1–2 of the five limitations (weight loss, exhaustion, slowness, weakness, and inactivity), respectively (12). “Weight loss” and “exhaustion” were assessed using two Kihon Checklist questions: “Have you lost ≥2 kg body weight in the past 6 months?” and “In the last 2 weeks, have you felt tired for no reason?” Individuals answering “yes” were defined as having “weight loss” and/or “exhaustion,” respectively. Walking speed was measured as time to walk 5 m at the usual pace, with “slowness” defined as <1.0 m/sec. Handgrip strength was assessed using a handheld dynamometer, with “weakness” defined by values <28 kg for men and <18 kg for women. Finally, inactivity was assessed by asking participants 1) “Do you engage in light-intensity exercise or calisthenics?” and 2) “Do you engage in exercise or sports activities?” Participants who answered “not at all” to both questions were defined as “inactive.”

### Stepping parameters assessed using Kinect^TM^

Participants were instructed to step for 20 s with their eyes open. This ST was conducted based on our unique protocol that included two modifications to the Fukuda stepping test (13): 1) participants stepped with their eyes open, and 2) the evaluation protocol was time-based rather than step count-based. Step cadence was determined by the individual and was not controlled. The Kinect^TM^ sensor was fixed using a tripod such that the sensor center was 1.0 m and 3.0 m from the floor and participants, respectively.

Step number (SN) and knee total movement distance (KMD) were evaluated during a 20-s ST using a Kinect^TM^ V2 infrared depth sensor (Microsoft Corporation, WA, US) as described previously (10, 14). During the final 10 s of the test, knee joint point coordinate data were recorded with the Kinect^TM^ depth sensor. It employs the “Time of Flight” technique, where an infrared projector emits pulse-modulated infrared light, determining the depth of the participant’s joints by analyzing the timing of the reflected light detected by the infrared camera. Motion analysis with Kinect^TM^ sensors is sufficiently accurate, with approximately 95% of valid measurement data used for accuracy verification within 100 mm error and approximately 88% within 40 mm standard deviation (15).

SN and KMD were calculated by processing knee joint coordinate data. For SN, smoothing was performed by applying a 9-point moving average filter to left and right knee joint coordinate data. Signals from the left and right knee joints were then added, and a 7-point low-differential filter was applied to convert them into velocity signals. Finally, these parameters were z-scored, and the zero-crossing points were extracted and defined as SN.

KMD was defined as the average total movement distance of both knee joints. First, joint coordinate point vectors of both knees at any given time (t) were defined as K^L^ (t) and K^R^ (t), respectively. Next, total movement distance in three-dimensional (3D) space, K_sum_^L^ and K^R^_sum_were calculated from equations i) and ii), respectively. Finally, KMD was calculated by averaging K^L^_sum_and K^R^_sum_R (iii). SN and KMD were corrected for sensor collection time and used for statistical analysis.

**Figure:**
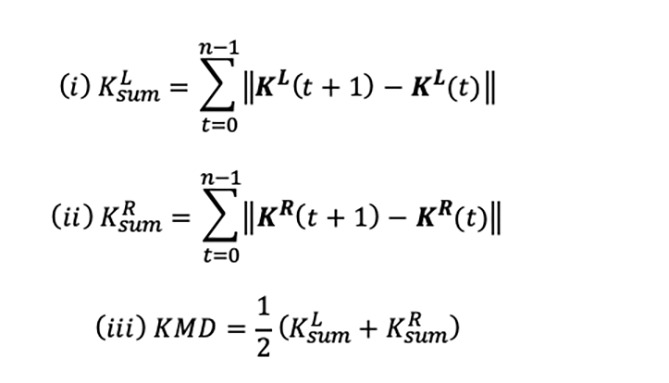


### Statistical analysis

All analyses were performed using IBM SPSS version 25.0 (IBM Corp., Armonk, New York, USA) and STATA 17.0 (StataCorp LLC, Texas, USA), with P-values <0.05 considered statistically significant.

First, Jonckheere–Terpstra trend tests (16, 17) were applied to analyze the dose-response relationships between the severity of frailty, SN, and KMD. Next, a binomial logistic regression model, with frailty phenotype as the dependent variable and SN and KMD as independent variables, was applied to construct a predictive model of frailty phenotype using the composite stepping parameters of SN and KMD. Finally, receiver operator characteristic analysis was applied to demonstrate the discriminative ability of SN and KMD for identifying frailty phenotype. Sensitivity and 1-specificity were plotted, and the AUC was calculated.

## Results

The characteristics of the study participants are presented in Table 1. The number (%) of participants with frailty and prefrailty were 51 (9.1) and 319 (56.7), respectively. Figure 1 shows that higher frailty severity was significantly associated with lower medians [interquartile ranges] of SN (robust, 1.81 [1.67–1.97]; prefrailty, 1.78 [1.63–1.92]; and frailty, 1.71 [1.53–1.82], p for trend <0.05) and KMD (robust, 0.49 [0.40–0.62]; prefrailty, 0.43 [0.34–0.53]; and frailty, 0.35 [0.25–0.44], p for trend <0.05). AUCs (95% confidence interval [CI]) for the frailty phenotype of the composite stepping parameter were 0.72 (0.64, 0.79), 0.73 (0.60, 0.86), and 0.73 (0.64, 0.81) for all participants, men, and women, respectively.

**Table 1.** Characteristics of study participants

	All n = 563	Men n = 218	Women n = 345
Demographics			
Age, years	79 [77–82]	79 [77–82]	79 [77–82]
Height, cm	153.9 [149.2–160.1]	162.6 [158.3–167.2]	150.3 [146.7–153.5]
Weight, kg	53.5 [46.9–61.5]	61.2 [56.0–67.6]	49.4 [44.7–54.4]
BMI, kg/m^2^	22.5 [20.4–24.8]	23.1 [21.6–25.2]	21.9 [19.8–24.4]
Medical history			
Hypertension	294 (52.2)	115 (52.8)	179 (51.9)
Stroke	28 (5.0)	14 (6.4)	14 (4.1)
Heart disease	106 (18.8)	45 (20.6)	61 (17.7)
Diabetes	78 (13.9)	45 (20.6)	33 (9.6)
Hyperlipidemia	196 (34.8)	57 (26.1)	139 (40.3)
Cognitive function			
MMSE-J, score	28 [26–29]	28 [25–29]	28 [26–29]
Lifestyle			
Smoker	23 (4.1)	13 (6.0)	10 (2.9)
Drinker	242 (43.0)	133 (61.0)	109 (31.6)
Frailty phenotype			
Frailty	51 (9.1)	17 (7.8)	34 (9.9)
Shrinking	75 (13.3)	38 (17.4)	37 (10.7)
Weakness	209 (37.1)	80 (36.7)	129 (37.4)
Exhaustion	175 (31.1)	59 (27.1)	116 (33.6)
Slowness	91 (16.2)	39 (17.9)	52 (15.1)
Low activity	62 (11.0)	21 (9.6)	41 (11.9)
Kinect^TM^ parameters			
Step number, steps/s	1.77 ± 0.23	1.71 ± 0.24	1.81 ± 0.22
KMD, m/s	0.46 ± 0.16	0.44 ± 0.15	0.48 ± 0.17

Note. The data are shown as median [interquartile range], mean ± standard deviation, or n (%). BMI: body mass index, MMSE-J: Mini Mental State Examination-Japanese, KMD: knee total movement distance. Smoker or drinker were defined as participants who had a history of such behaviors.

**Figure 2.**
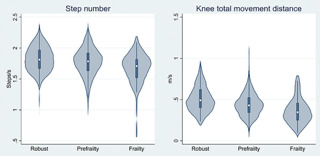
Dose-response relationships between frailty severity, step number (SN), and knee total movement distance (KMD)

Note: The figure shows the data distribution of SN and KMD based on frailty severity categories by using a violin plot. The width of the density curves corresponds to the frequency of the data. The box plots drawn in the center of each density curve show the first and third quartile ends, and the central circle shows the median.

## Discussion/Conclusion

This study quantifies the discriminative ability of stepping performance during a 20-s ST using the Kinect^TM^ infrared depth sensor for identifying frailty phenotype in a cross-section sample. Several mobility assessment instruments, such as gait speed and TUG tests, have been reported to discriminate frailty phenotype (gait speed, AUC [95% CI]: 0.73 [0.65, 0.80]; TUG, AUC [95% CI]: 0.76 [0.68, 0.83]) (18); however, the discriminative ability of the composite stepping parameter assessed by Kinect^TM^ may provide added benefit in the assessment such as providing information regarding balance function (5, 10) or identifying potentially negative movement compensations as observed with an increased torso angle during the chair-stand test when using Kinect^TM^ (19) or lower SN during the ST (10) in discriminating assisted-living from independent living older adults. These results indicate that the composite stepping parameter using Kinect^TM^ may have a clinically acceptable discriminatory performance as a simple screening tool for frailty phenotype.

Stepping performance can be accurately quantified using a 3D motion capture system; however, such systems are expensive and, hence, limited for use in primary care or in-home settings. Kinect^TM^ may be able to fill this gap as an inexpensive, portable, and easy-to-set-up system in such settings (5). In addition, Kinect-based exergames have been reported to be as beneficial as traditional structured exercises (20) in improving physical function. In the future, performance analysis with the 20-s ST and Kinect^TM^ may provide integrated assessment towards designing and assessing interventions, as well as developing practical and sustainable frailty coping strategies in primary care and at-home environments.

As this study was conducted with older adults living in metropolitan areas in Japan, there may be limitations in the generalizability of results to other regions or populations. This study demonstrates the discriminative ability of the ST and Kinect^TM^-based composite stepping parameters as a screening tool for frailty. However, it is unclear whether it 1) predicts the onset of frailty or subsequent important health outcomes and 2) is acutely responsive to care interventions. These remain important future longitudinal and interventional study questions that will clarify these points and the clinical utility of Kinect^TM^-based composite stepping parameters towards assessing frailty, its onset, and remediation interventions.

In summary, stepping parameters derived from a 20-s ST assessed using a Kinect^TM^ infrared depth sensor can discriminate frailty, indicating its utility as an inexpensive and widely available screening tool for frailty. Longitudinal studies and studies involving different sample populations will strengthen the generalizability and clinical utility of this tool.

## Data Availability

Research data are not shared.
